# Go Local: Innovating Local Health Departments' Engagement on Alzheimer’s

**DOI:** 10.1093/geroni/igaa057.186

**Published:** 2020-12-16

**Authors:** John Shean, Molly French

**Affiliations:** Alzheimer's Association, Washington, District of Columbia, United States

## Abstract

The aging Baby Boom generation is a major force behind projected increases in the prevalence of Alzheimer’s, which is expected to grow from 5.8 million (2020) to 13.8 million (2050). Local health departments play a major role connecting people living with dementia (and their caregivers) to services, supports, and education, and to ensure safe, accessible environments where they can flourish. From September 2019-July 2020, two local health departments (from San Diego County and the City of Boston) participated in a yearlong collaborative pilot project with the Alzheimer’s Association to advance cognitive health, dementia, and caregiving issues in their local jurisdictions. The National Association of County and City Health Officials (NACCHO) provided expert guidance and input throughout the collaboration. As part of the project, the local health departments: scanned their current work on healthy aging, identified existing data sources, and examined how existing healthy equity initiatives can apply to cognitive health, dementia, and caregiving issues. Action plans were developed, with a focus on policy mechanisms to initiate and sustain these projects and workforce development initiatives. Plans corresponded to actions of the Healthy Brain Initiative Road Map, helping elevate recommendations to change systems, policies, and environments. In fall 2020, LHDs will be able to use best practices, case studies, and tools developed from San Diego’s and Boston’s pilots to address Alzheimer’s as a chronic condition. The tools will help them engage health officials, develop action plans, and train the public health

